# Improving the GRACE Kinematic Precise Orbit Determination Through Modified Clock Estimating

**DOI:** 10.3390/s19194347

**Published:** 2019-10-08

**Authors:** Xingyu Zhou, Weiping Jiang, Hua Chen, Zhao Li, Xuexi Liu

**Affiliations:** 1 GNSS Research Center, Wuhan University, Wuhan 430079, China; zhouxygps@whu.edu.cn (X.Z.); wpjiang@whu.edu.cn (W.J.); 2 School of Geodesy and Geomatics, Wuhan University, Wuhan 430079, China; xuexiliu@whu.edu.cn; 3 Department of Land Surveying and Geo-Informatics, The Hong Kong Polytechnic University, 181 Chatham Road South, Hung Hom, Kowloon 999077, Hong Kong; zhao.li@whu.edu.cn

**Keywords:** clock estimating, GRACE, kinematic precise orbit determination, PPP-AR, GPS

## Abstract

Utilizing global positioning system (GPS) to determine the precise kinematic orbits for the twin satellites of the Gravity Recovery and Climate Experiment (GRACE) plays a very important role in the earth’s gravitational and other scientific fields. However, the orbit quality is highly depended on the geometry of observed GPS satellites. In this study, we propose a kinematic orbit determination method for improving the GRACE orbit quality especially when the geometry of observed GPS satellites is weak, where an appropriate random walk clock constraint between adjacent epochs is recommended according to the stability of on-board GPS receiver clocks. GRACE data over one month were adopted in the experimental validation. Results show that the proposed method could improve the root mean square (RMS) by 20–40% in radial component and 5–20% in along and cross components. For those epochs with position dilution of precision (PDOP) larger than 4, the orbits were improved by 50–70% in radial component and 17–50% in along and cross components. Meanwhile, the Allan deviation of clock estimates in the proposed method was much closer to the reported Allan deviation of GRACE on-board oscillator. All the results confirmed the improvement of the proposed method.

## 1. Introduction

The twin satellites of the Gravity Recovery and Climate Experiment (GRACE) mission were launched on 17 March 2002 at an altitude of approximately 500 km for making detailed measurements of Earth’s gravity [1], where the precise orbits play a very important role. In order to determine the precise orbits, both GRACE satellites carry a dual-frequency BlackJack GPS receiver with high-quality oscillators. It is reported that an accuracy of a few centimeters could be achieved for GRACE orbits by GPS [2].

Currently, there are two typical methods to obtain the GRACE precise orbits using GPS including reduced-dynamic precise orbit determination (RDPOD) [3] and kinematic precise orbit determination (KPOD) [4]. The RDPOD could achieve stable results with an accuracy of 1–2 cm [5], where not only GPS data but also dynamic force models are used. Whereas, in the KPOD only GPS data are used. Compared to RDPOD, the orbits obtained by KPOD are independent with dynamic force models and are considered as an alternative for gravity recovery. It is also reported that an absolute accuracy of centimeter level in space is achievable by KPOD [6,7]. Moreover, KPOD has been successfully applied to other scientific researches [8,9,10].

However, the orbit quality by KPOD is highly depended on the observed GPS satellite geometry. When the geometry of observed GPS satellites is not strong enough, the orbits would become unreliable or even unusable at those epochs. Fortunately, ultra stable oscillators (USO) with stabilities of $1–3\times 10^{-13}$ for averaging times from 1 s to 1000 s are equipped onboard [11], which provide opportunities to improve this situation. In the normal procedure of KPOD, the receiver clock offsets are estimated as epoch-wise parameters without considering the highly stable feature of onboard oscillators. In order to solve this problem, the receiver clocks are suggested to be estimated as piece-wise linear parameters in the estimations with least squares (LS) solution [12,13]. It is reported that the robustness and accuracy of satellite trajectory could be improved by about 40% in the radial direction and 7% in the along-track and cross-track directions, when a 60-s piecewise linear clock model is utilized [13]. Meanwhile, a three-order polynomial model is also available for this situation where the receiver clocks are expressed by offset, frequency offset and frequency drift based on the extended Kalman filter (EKF) solution [14,15]. Comparable results to the linear clock model could be achieved by the polynomial model.

In this study, we provide an alternative KPOD method based on the relationship between consecutive clock offset estimates. The receiver clock offsets are estimated as epoch-wise parameters with an appropriate random walk (RW) constraint between adjacent epochs. This method is easy to realize and could improve the orbit accuracy and reliabilities greatly, especially in poor geometry conditions. Meanwhile, the performance of this method is assessed in a simulated real-time situation to investigate its potential in improving accuracy and shortening converge time. The study is organized as follows. Section 2 presents the feasibility of estimating receiver clocks as RW parameters based on the stability of onboard USO and the KPOD algorithm in detail. Experiment design and data processing strategies are introduced in Section 3. The results and analysis are included in Section 4. Finally, conclusions are discussed in Section 5.

## 2. Methodology

### 2.1. GPS Observation Model

The observation equation of ionosphere-free (IF) combination in precise kinematic orbit determination is as follows [16]:

(1) $$\begin{array}{c} P_{IF}=\rho_{r}^{s}+c\left(\delta t_{r}-\delta t^{s}\right)+\delta_{rel,r}^{s}+\delta_{windup,r}^{s}+\left(\delta_{pco,r}+\delta_{pcv,r}\right)\\ +\left(\delta_{pco}^{s}+\delta_{pcv}^{s}\right)+\varepsilon_{P_{IF}}, \end{array}$$

(2) $$\begin{array}{c} L_{IF}=\rho_{r}^{s}+c\left(\delta t_{r}-\delta t^{s}\right)+\delta_{rel,r}^{s}+\delta_{windup,r}^{s}+\left(\delta_{pco,r}+\delta_{pcv,r}\right)\\ +\left(\delta_{pco}^{s}+\delta_{pcv}^{s}\right)+\lambda_{IF}B_{IF}+\varepsilon_{L_{IF}}, \end{array}$$

where $P_{IF}$ and $L_{IF}$ are code and carrier phase *IF* observations; $\rho_{r}^{s}$ means geometric distance between GPS satellite s and low earth orbit (LEO) satellite receiver r; c means speed of light; $\delta t_{r}$ and $\delta t^{s}$ are receiver and satellite clock errors, respectively; $\delta_{rel,r}^{s}$ means relativistic delay; $\delta_{windup,r}^{s}$ means phase wind-up delay; $\delta_{pco,r}$ and $\delta_{pcv,r}$ are phase center offset (PCO) and phase center variation (PCV) of LEO receiver; $\delta_{pco}^{s}$ and $\delta_{pcv,r}$ are PCO and PCV of GPS satellites; $\lambda_{IF}$ means wavelength of *IF* combination; $B_{IF}$ means float ambiguity of *IF* combination and $\varepsilon_{P_{IF}}$ and $\varepsilon_{L_{IF}}$ are un-modeled code and carrier phase errors. The troposphere delay is neglected as the LEO satellites fly above the troposphere. Meanwhile, due to the on-board GPS receiver PCV is not available at present, it will be neglected in the actual data processing.

In LS solution, all observations over the whole processing period are gathered to the normal equation (NEQ). Assuming the observation equations are,

(3) v=Ax+l, P,

where v is the observation residuals, A is the design matrix, x is the vector of estimates, l is the observation minus computed vector and P is the weight matrix. Then corresponding normal equation can be introduced,

(4) $$\{\begin{array}{c} Nx=b\\ N=A^{T}PA\\ b=A^{T}Pl \end{array},$$

where N is the normal equation matrix and b represents the right side of the normal equation. In order to clarify the description, only the position and clock parameters are considered, then the normal equation for two adjacent epochs $t_{i}$ and $t_{i}+\tau$ is written as,

(5) $$\left[\begin{array}{cccccccc} N_{ii}^{11} & N_{ii}^{12} & N_{ii}^{13} & N_{ii}^{14} & 0 & 0 & 0 & 0\\ N_{ii}^{21} & N_{ii}^{22} & N_{ii}^{23} & N_{ii}^{24} & 0 & 0 & 0 & 0\\ N_{ii}^{31} & N_{ii}^{32} & N_{ii}^{33} & N_{ii}^{34} & 0 & 0 & 0 & 0\\ N_{ii}^{41} & N_{ii}^{42} & N_{ii}^{43} & N_{ii}^{44} & 0 & 0 & 0 & 0\\ 0 & 0 & 0 & 0 & N_{i+1,i+1}^{11} & N_{i+1,i+1}^{12} & N_{i+1,i+1}^{13} & N_{i+1,i+1}^{14}\\ 0 & 0 & 0 & 0 & N_{i+1,i+1}^{21} & N_{i+1,i+1}^{22} & N_{i+1,i+1}^{23} & N_{i+1,i+1}^{24}\\ 0 & 0 & 0 & 0 & N_{i+1,i+1}^{31} & N_{i+1,i+1}^{32} & N_{i+1,i+1}^{33} & N_{i+1,i+1}^{34}\\ 0 & 0 & 0 & 0 & N_{i+1,i+1}^{41} & N_{i+1,i+1}^{42} & N_{i+1,i+1}^{43} & N_{i+1,i+1}^{44} \end{array}\right]\left[\begin{array}{c} x\left(t_{i}\right)\\ y\left(t_{i}\right)\\ z\left(t_{i}\right)\\ \delta t_{r}\left(t_{i}\right)\\ x\left(t_{i}+\tau \right)\\ y\left(t_{i}+\tau \right)\\ z\left(t_{i}+\tau \right)\\ \delta t_{r}\left(t_{i}+\tau \right) \end{array}\right]=\left[\begin{array}{c} b_{i}\\ b_{i+1} \end{array}\right],$$

where x, y and z are the linear expansion of $\rho_{r}^{s}$ in Equations (1) and (2), $N_{ii}$ and $N_{i+1,i+1}$ are corresponding parts in normal equation matrix N for epoch $t_{i}$ and $t_{i}+\tau$ separately, and $b_{i}$ and $b_{i+1}$ are also corresponding parts with respect to b. It can be noticed that no connections between two adjacent epochs are found if the ambiguity parameters are not considered. That is also the reason that at least four satellites are needed to determine the position.

### 2.2. On-Board GPS Clock Constraints

By considering that the Allan deviation of GRACE USO was about $1–3\times 10^{-13}$ [11] and the sampling of GRACE data was only 10 s, we assumed that the GRACE on-board receiver clock could be estimated as random walk parameters. In the LS processing, it can be written as,

(6) $$E\left(\delta t_{r}\left(t_{i}+\Delta t\right)-\delta t_{r}\left(t_{i}\right)\right)=\Delta ,$$

where the consecutive clock offset estimates are subtracted directly and expected to be equal to a constant Δ if Δt is less than 60 s [13]. The variation between two consecutive clock estimates can be set to the value of Allan deviation of the onboard USO times the sampling. Therefore, Δ is about $1–3\times 10^{-12}\;s$ (0.3–0.9 cm) when Δt is 10 s. It is far less than the level of clock offset variations in experience and fully overwhelmed by receiver clock noises, thereby a proper random walk model can involve it and larger noises are possibly avoided. Thus in Equation (6), the difference of two adjacent $\delta t_{r}$ parameters can be totally represented by this random walk model. Suppose Δ is expected to be zero in Equation (6), then clock offset constraints can be imposed directly as pseudo observation equations in the unit of meters,

(7) $$\begin{array}{c} \begin{array}{c} \delta t_{r}\left(t_{i}+\tau \right)-\delta t_{r}\left(t_{i}\right) \end{array} \end{array}=\Delta =0,\;\begin{array}{c} \begin{array}{c} p_{\Delta }=\sigma_{0}^{2}/D_{\Delta } \end{array} \end{array},$$

where $p_{\Delta }$ is the power of Δ, and $D_{\Delta }$ is the variance. Thus the essential problem is to determine the value of $D_{\Delta }$. In this study, we assumed it as:

(8) $$\begin{array}{c} D_{\Delta }=c\cdot \mathrm{AllanDeviation}\cdot \Delta t\cdot \mathrm{factor} \end{array},$$

where c is the speed of light; AllanDeviation is the average of USO Allan deviation ($2\times 10^{-13}$), which means the variation of clock offsets in unit time; Δt was set to the sampling interval τ (10 s) and the factor for larger tolerance was set to 8 according to our experience. In order to merge the pseudo observation into the normal equations [17], Equation (7) could be rewritten as,

(9) $$\left[\begin{array}{cccccccc} 0 & 0 & 0 & -1 & 0 & 0 & 0 & 1 \end{array}\right]\left[\begin{array}{c} x\left(t_{i}\right)\\ y\left(t_{i}\right)\\ z\left(t_{i}\right)\\ \delta t_{r}\left(t_{i}\right)\\ x\left(t_{i}+\tau \right)\\ y\left(t_{i}+\tau \right)\\ z\left(t_{i}+\tau \right)\\ \delta t_{r}\left(t_{i}+\tau \right) \end{array}\right]=0.$$

The normal equation matrix for Equation (9) could be written as,

(10) $$N_{\Delta }=\left[\begin{array}{c} 0\\ 0\\ 0\\ -1\\ 0\\ 0\\ 0\\ 1 \end{array}\right]p_{\Delta }\left[\begin{array}{cccccccc} 0 & 0 & 0 & -1 & 0 & 0 & 0 & 1 \end{array}\right]=\left[\begin{array}{cccccccc} 0 & 0 & 0 & 0 & 0 & 0 & 0 & 0\\ 0 & 0 & 0 & 0 & 0 & 0 & 0 & 0\\ 0 & 0 & 0 & 0 & 0 & 0 & 0 & 0\\ 0 & 0 & 0 & p_{\Delta } & 0 & 0 & 0 & -p_{\Delta }\\ 0 & 0 & 0 & 0 & 0 & 0 & 0 & 0\\ 0 & 0 & 0 & 0 & 0 & 0 & 0 & 0\\ 0 & 0 & 0 & 0 & 0 & 0 & 0 & 0\\ 0 & 0 & 0 & -p_{\Delta } & 0 & 0 & 0 & p_{\Delta } \end{array}\right].$$

After combining Equations (5) and (10), the final normal equation could be derived as,

(11) $$\left[\begin{array}{cccccccc} N_{ii}^{11} & N_{ii}^{12} & N_{ii}^{13} & N_{ii}^{14} & 0 & 0 & 0 & 0\\ N_{ii}^{21} & N_{ii}^{22} & N_{ii}^{23} & N_{ii}^{24} & 0 & 0 & 0 & 0\\ N_{ii}^{31} & N_{ii}^{32} & N_{ii}^{33} & N_{ii}^{34} & 0 & 0 & 0 & 0\\ N_{ii}^{41} & N_{ii}^{42} & N_{ii}^{43} & N_{ii}^{44}+p_{\Delta } & 0 & 0 & 0 & -p_{\Delta }\\ 0 & 0 & 0 & 0 & N_{i+1,i+1}^{11} & N_{i+1,i+1}^{12} & N_{i+1,i+1}^{13} & N_{i+1,i+1}^{14}\\ 0 & 0 & 0 & 0 & N_{i+1,i+1}^{21} & N_{i+1,i+1}^{22} & N_{i+1,i+1}^{23} & N_{i+1,i+1}^{24}\\ 0 & 0 & 0 & 0 & N_{i+1,i+1}^{31} & N_{i+1,i+1}^{32} & N_{i+1,i+1}^{33} & N_{i+1,i+1}^{34}\\ 0 & 0 & 0 & -p_{\Delta } & N_{i+1,i+1}^{41} & N_{i+1,i+1}^{42} & N_{i+1,i+1}^{43} & N_{i+1,i+1}^{44}+p_{\Delta } \end{array}\right]\left[\begin{array}{c} x\left(t_{i}\right)\\ y\left(t_{i}\right)\\ z\left(t_{i}\right)\\ \delta t_{r}\left(t_{i}\right)\\ x\left(t_{i}+\tau \right)\\ y\left(t_{i}+\tau \right)\\ z\left(t_{i}+\tau \right)\\ \delta t_{r}\left(t_{i}+\tau \right) \end{array}\right]=\left[\begin{array}{c} b_{i}\\ b_{i+1} \end{array}\right].$$

Notice that the values of b in Equations (5) and (11) are the same, as the expectation of the constraint is zero. Therefore, with the RW constraints, we could limit the variations of clock offsets in consecutive epochs tightly, making them closer to real USO variation trends.

## 3. Data and Processing Strategies

In our study, the GRACE data of the twin satellites, GRACE-A and GRACE-B, from 1 to 28 February 2007 were adopted, including satellite-borne GPS observation data (GPS1B), attitude data (SCA1B) and reference satellite orbit products (NAV1B) provided by GeoForschungsZentrum (GFZ). The NAV1B product is of high accuracy and has been widely used for reference orbits. All data above can be found in the GFZ open website (http://isdc-old.gfz-potsdam.de/). Meanwhile, in order to evaluate the performance of the proposed method, we conducted three experiments in precise point positioning (PPP) solution [18]. In the first experiment, we calculated a KPOD result of float solution with and without the RW constraints and verified the orbit accuracies, especially when the geometry was weak. In the second experiment, to validate the promotion of the RW constraints in PPP ambiguity resolution (PPP-AR) solution, comparisons of orbits with and without RW were investigated. Finally, an epoch-wise LS experiment was conducted to investigate the potential of this method in real-time applications.

Experimental data processing strategies are shown in Table 1. All experiments were carried out with dual-frequency code and phase observations. To eliminate the first order impact of ionosphere, the ionosphere-free linear combinations of $P_{IF}$ and $L_{IF}$ were used separately. The impacts of troposphere delay were neglected since LEO satellites fly over the troposphere. The windup corrections were applied using the Wu model [19]. Receiver antenna PCO was corrected by GFZ level 1B products, while PCV was not corrected as lacking of released empirical models. Meanwhile, the attitude products (SCA1B) were used for the transformation between satellite body-fixed frame and celestial reference frame. Moreover, relativity corrections were applied for all GPS observations [20,21].

International GNSS Service (IGS) final orbits and final 30-s clocks were fixed in the PPP processing. The GPS PCO and PCV were taken from the IGS antenna file IGS08.atx (week 1930). For stochastic models, we used an elevation dependent model and the relative weighting of code and carrier phase was 1/100^2^. Data sampling interval was 10 s and those observations below 0° were excluded. In the post-processing, all observations were added to an NEQ and the parameter elimination and recovery method [22] was used, where all estimates were solved at one time. While for epoch-wise solutions, they were solved epoch to epoch, and adopted for simulated real-time experiments.

In the following, three experiments were designed to assess the performance of this method in the PPP float mode, PPP-AR mode and simulated real-time mode, respectively. In the PPP-AR mode, the uncalibrated phase delays (UPDs) for GPS satellites were calculated with a selected IGS global network of 164 sites and had been proved of high accuracy for ground sites [23].

## 4. Results and Analysis

### 4.1. PPP Float Results

**Figure 1:**
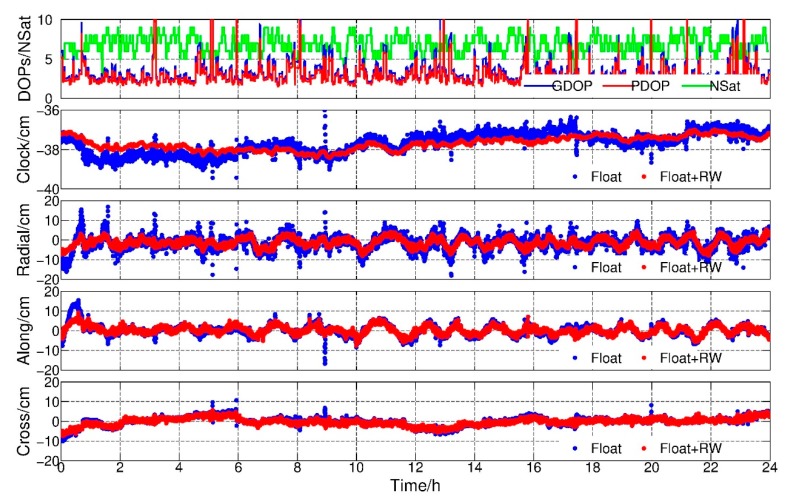
PDOP, GDOP, number of satellites, clock offsets and radial-along-cross residuals, float solution and float + random walk (RW) solution, Gravity Recovery and Climate Experiment (GRACE)-B, 1 February 2017.

In this study, all results were compared to the reduced-dynamic orbit of GRACE Level 1B products (NAV1B) provided by GFZ for assessing the orbit quality. We analyzed GRACE-B results as an example since the observation condition of GRACE-A was better than GRACE-B during the experiments. Figure 1 shows the position dilution of precision (PDOP), geometric dilution of precision (GDOP), number of observed satellites, receiver clock offsets and orbit residuals in radial, along and cross components with respect to the reference orbit. The blue and red dots are float solutions with and without RW constraints, respectively. As shown in the top subfigure, the average PDOP and GDOP values were 3.20 and 3.64, respectively. Among them, 14.53% and 20.16% PDOPs were over 4. When PDOPs were less than 2, a stable cm-level positioning result could be achieved using the PPP method, whereas, the positioning results might fluctuate when the PDOPs were larger than 4. It suggests that the KPOD could not be guaranteed in poor geometry conditions.

In the second subfigure, there were obvious jumps in the high PDOP value epochs, i.e., 9:00, and the deviation of the estimated float clock offset series was 5.21 cm. The jumps implied that the receiver clock estimates probably absorbed part of the positioning errors. However, after applying the RW constraints between the two adjacent epochs, the receiver clock estimates became smoother even in high DOP value epochs. The deviation of the receiver clock estimates was 3.35 cm, and conducted 35.75% improvements with respect to the float solution. Meanwhile, the differences between the kinematic orbit and reference orbit were shown in radial-, along- and cross-track directions. It could be found that the kinematic orbits became smoother and more convergent after constraints applied.

**Figure 2:**
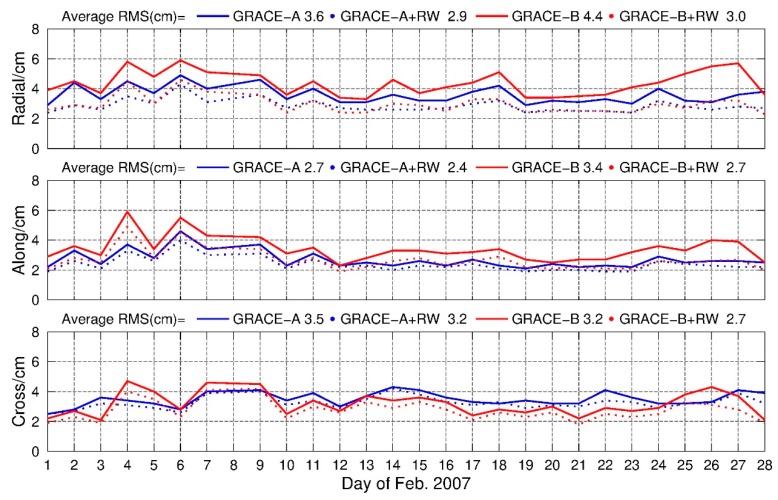
Daily RMS of GRACE-A and GRACE-B, float solution with and without RW constraints, 1–28 February 2017.

Figure 2 is the daily root mean square (RMS) of the orbits for February 2007, and the average RMS are summarized in Table 2. An improvement of 19.44%, 11.11% and 8.57% of GRACE-A in radial, along and cross components can be found, and the corresponding improvements for GRACE-B were 31.82%, 20.59% and 15.63%, respectively. The greatest improvement was found in the radial direction due to the highly correlation between the radial component and the receiver clock parameter, which was consistent with the study of Weinbach and Schön [13]. In addition, from Figure 1, it could be inferred that the main advantage of imposing RW constraints was avoiding large jumps when the geometry of observed GPS satellites was weak.

**Figure 3:**
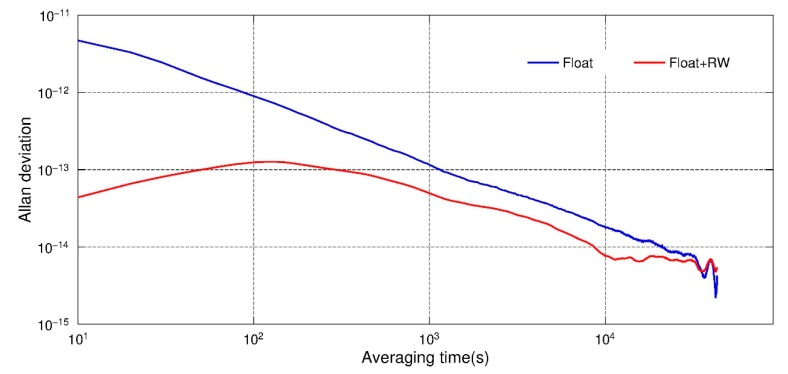
Allan deviation of clock offset estimates, float solution with and without RW constraints, GRACE-B, 1 February 2007.

The Allan deviation of the estimated clock parameters could also be an index to assess the performance of this new method. Figure 3 shows the Allan deviation of clock offset estimates of the GRACE-B satellite in 1 February 2007. It could be found that the Allan deviation calculated from PPP float solutions shows a near linear decline in a logarithmic axis and no short-term stability is shown. Whereas, after the constraints were applied, the value remained around 10^−13^ in 1–1000 s, which is consistent with the study of Dunn et al. [11]. This indicates that after applying clock constraints, the clock offset estimates were closer to the real clock performance. After 1000 s, the trend of clock estimates also declined similar to the float solution. It is because the systematic error actually domains the Allan deviation [13].

**Figure 4:**
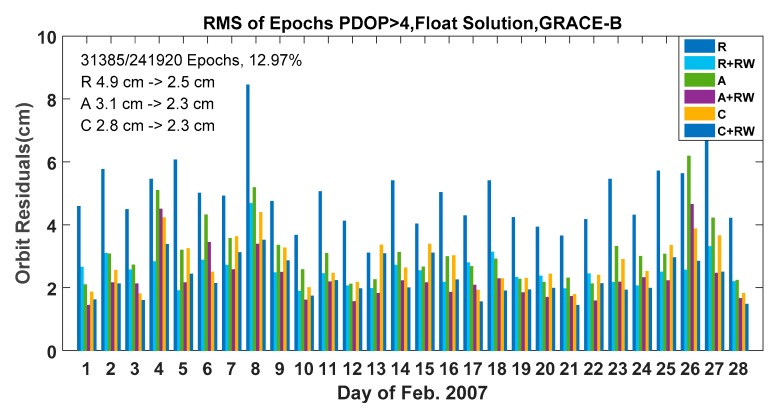
RMS for Epochs PDOP greater than 4, float solution with and without RW constraints, GRACE-B, 1–28 February 2017.

In order to confirm the improvements in poor geometry conditions of this method, all orbit residuals of epochs with PDOPs greater than 4 were selected for further evaluation. There were 241,920 epochs in estimation, and about 12.97% (31,385) epochs had a PDOP larger than 4. Figure 4 shows the RMS for epochs with PDOP larger than 4 for each day in February 2007. It can be found the average RMS of the radial, along and cross residuals were improved from 4.9 cm, 3.1 cm, 2.8 cm to 2.5 cm, 2.3 cm and 2.3 cm respectively. These results confirmed that the random walk constraints could effectively refine the positioning results in poor observation conditions.

### 4.2. PPP-AR Results

It is well known that PPP-AR can significantly improve the accuracy of PPP-based kinematic orbits [24]. Thereby, the impacts of random walk constraints on PPP-AR solutions were investigated. In this section, the integer ambiguity resolution was conducted with well calculated UPD products. A set of UPDs were estimated using 164 global stations on the ground, where the IGS final products used were the same with the KPOD processing.

**Figure 5:**
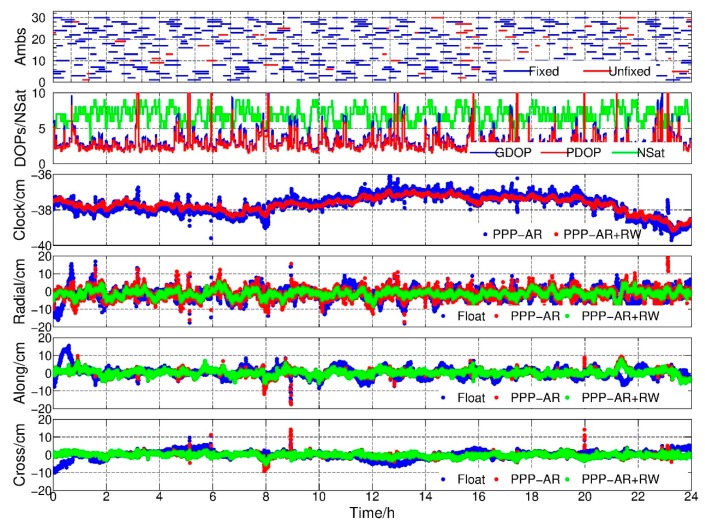
Ambiguities, PDOP, GDOP, number of satellites, clock offsets and radial-along-cross residuals, float, precise point positioning ambiguity resolution (PPP-AR) and PPP-AR + RW, GRACE-B, 1 February 2007.

Similarly, Figure 5 shows the PPP-AR solution with and without random walk constraints for GRACE-B in 1 February 2007. The ambiguities of each GPS satellite, PDOP, GDOP, number of satellites, receiver clock offsets and orbit residuals in radial, along and cross components with respect to reference orbit are shown from top to bottom. In the top of Figure 5, the blue lines represent those fixed ambiguity arcs, while the red lines are ambiguity arcs that fail to be fixed to integers. We found that most unfixed ambiguities were related to those epochs with larger PDOPs. The DOPs for PPP-AR solution were all the same as that for float solution because of the same observations. It also can be found the clock offset estimates were more convergent, and almost no obvious jumps were exhibited after the RW constraints were applied. Statistical results show that the RMS of clock estimates was reduced from 5.8 cm to 4.5 cm, which improved by about 22.41%.

For positioning results, the blue, red and green dot lines meant the float solution, PPP-AR solution and PPP-AR solution with random walk constraints, respectively. It can be found that the PPP-AR solutions with RW constraints were much more stable than the other two solutions, especially in those epochs with large DOPs. However, we also found for some epochs, the PPP-AR solutions were even a little worse than the float solution, such as near 9:00 and 20:00. This might be caused by some wrongly fixed ambiguities arcs. Nevertheless, after applying the RW constraints, the sharp fluctuations were reduced, and the positioning accuracy in radial component improved greatly, which confirmed the improvements caused by the RW constraints.

**Figure 6:**
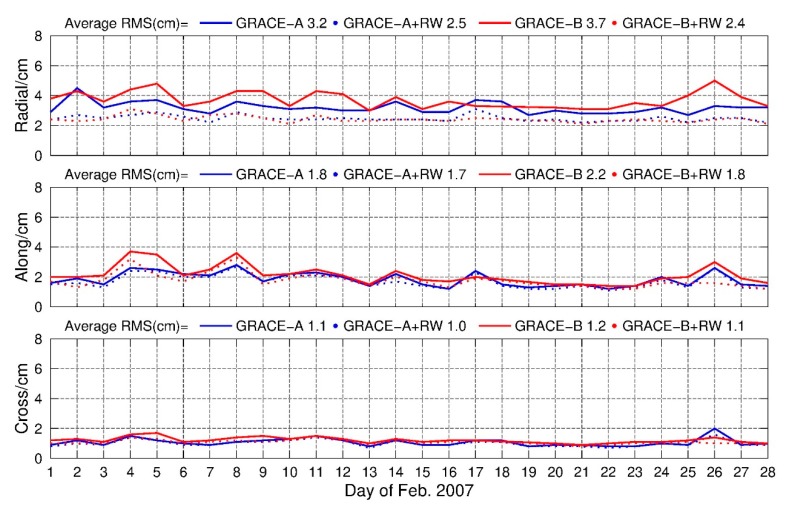
Daily RMS of GRACE-A and GRACE-B, PPP-AR solution with and without RW constraints, 1–28 February 2007.

Furthermore, Figure 6 shows the RMS of the PPP-AR solution in radial, along and cross components with and without RW constraints and the improvement ratios are illustrated in Table 3. In general, the average RMS of PPP-AR solution with RW constraints were less than 3 cm in radial component and 2 cm in along and cross components, and it was confident that the 3DRMS were less than 4 cm, which was considerably accurate for LEO KPOD results. The improvements of this method in radial component were 21.88% and 31.82% for GRACE A and GRACE B in statistics, respectively. Therefore, the new method was recommended in GRACE PPP-AR solutions.

**Figure 7:**
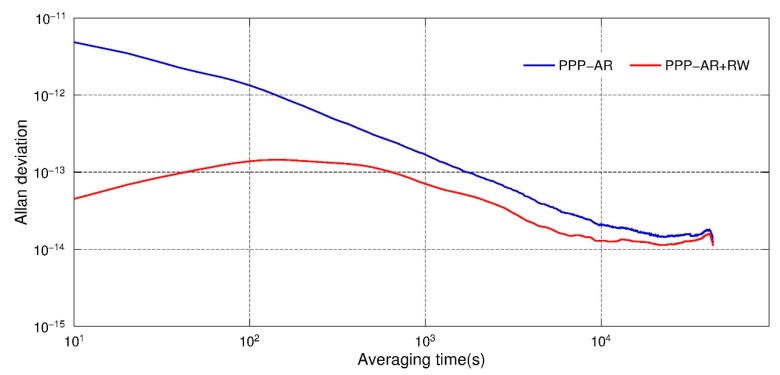
Allan deviation of clock offset estimates, PPP-AR solution with and without RW constraints, GRACE-B, 1 February 2007.

Similarly, the Allan derivation of clocks derived from PPP-fixed solutions were calculated and analyzed. The results are shown in Figure 7. It can be found that after applying the random walk constraints, the Allan derivation derived from clock estimates were much closer to the reported value 10^−13^. Meanwhile, statistical analysis was also carried out for those epochs PDOPs larger than 4 in February 2007. The average RMS for those epochs with PDOP greater than 4 could be reduced from 7.2 cm, 3.4 cm and 2.9 cm in radial, along and cross directions to 2.1 cm, 2.0 cm and 1.5 cm after RW constraints applied, respectively. These results confirmed the effects of this method in the PPP-AR solution.

### 4.3. Simulated Real-Time PPP Results

**Figure 8:**
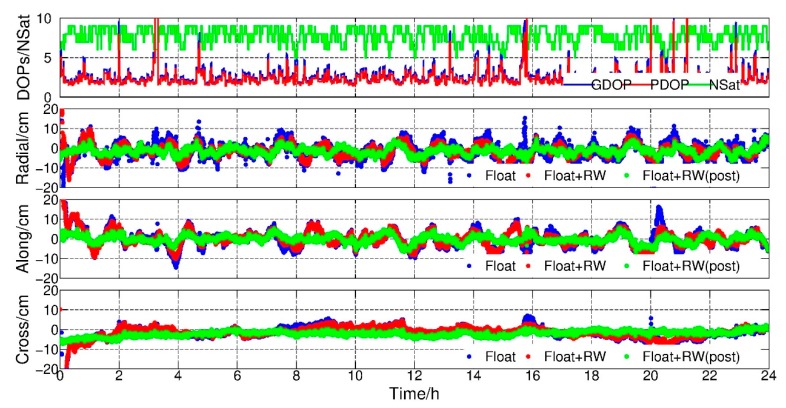
PDOP, GDOP, number of GPS satellites, radial-along-cross residuals, float, float + RW and float + RW (post-processing), GRACE-A, 1 February 2007.

**Figure 9:**
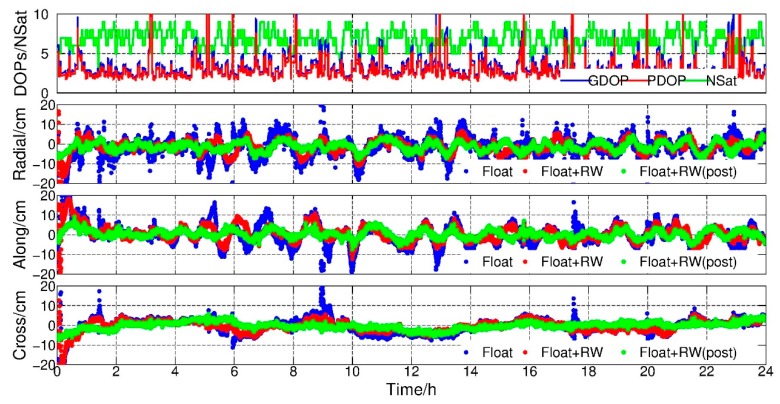
PDOP, GDOP, number of GPS satellites, radial-along-cross residuals, float, float + RW and float + RW (post-processing), GRACE-B, 1 February 2007.

The purpose of this experiment was to assess the potential of this new method in the real-time KPOD mode. In order to exclude the negative effects caused by the biases of GPS orbits and clocks, the IGS final orbits and clocks were adopted in the experiments. To evaluate the performance of the method, accuracy and converge time were also selected as two main indices. Similarly, the data of GRACE-A and GRACE-B satellites in 1 February 2007 were adopted. Figure 8 and Figure 9 show the PDOP, GDOP, number of satellites and orbit residuals in radial, along and cross components with respect to the reference orbits, respectively. For comparison, three positioning results are shown in the poisoning parts in Figure 8 and Figure 9. The first was normal epoch-wise float PPP results (blue dots), the second was the epoch-wise PPP results with random walk constraints (red dots) and the third was the reference one derived from post PPP model where all parameters were solved in the final normal equations (green dots).

From Figure 8 and Figure 9, we can see that the jumps were always found in those epochs with large DOP values. Similarly, after applying RW constraints, the orbit became much smoother and no big jumps occur. The average RMS of epoch-wise PPP float solution with and without RW constraints were calculated and shown in Table 4, where improvements of 26.83%, 21.95% and 16.00% for GRACE-A and 46.43%, 36.54% and 32.35% for GRACE-B in radial, along and cross directions could be found, respectively. Meanwhile, the solutions with RW constraints were comparable to the post-processing results after convergence.

**Figure 10:**
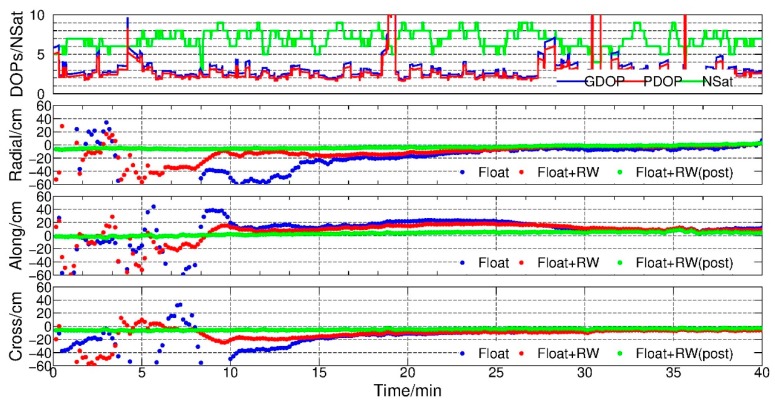
DOPs, number of GPS satellites, radial-along-cross residuals, float, float + RW and float + RW (post-processing), GRACE-B, 00:00–00:40, 1 February 2007.

In order to investigate whether the method could shorten the converging time, the converge time was defined as the time the residuals of 20 consecutive epochs fall to less than 20 cm in this study. We rescaled Figure 9, and only the first 40 min are shown in Figure 10. It can be seen that after applying the RW constraints, the converge time was shortened greatly. It took only about 10 min to achieve convergence while the normal PPP needed about 15 min. However, after 30 min, both methods could achieve a very similar accuracy level. Table 5 shows the residual details at 10 min and 30 min in addition.

To make it more clear, data of GRACE-B from 1 to 28 February were processed with 12 arcs each day (2 h per arc), and the average converge time of the float solution with and without RW constraints were 578.2 s and 868.5 s respectively, which shows a reduction of 290.3 s (near 5 min). It could be inferred that the constraints strengthened the normal equation and shortened converge time in initialization.

## 5. Conclusions

In this study, we introduced a modified receiver clock estimating method by imposing appropriate random walk constraints between two adjacent epochs based on the relationship of clock offset estimates. The performance of this new method was investigated by utilizing one-month data of GRACE twin satellites in the experiments. The results show that, after applying the clock constraints, the RMS of PPP float and PPP-AR KPOD orbits could improve 20–40% in a radial component and 5–20% in along and cross components. For those epochs with a PDOP greater than 4, the orbits were improved by 50–70% and 17–50% in radial and other directions. The average RMS in all components was found to be less than 3 cm. Meanwhile, after applying the clock constraints, the Allan deviation of estimated clock offset parameters were more stable in 1000 s, and was closer to the reported Allan deviation of GRACE on-board USO. Furthermore, to investigate the potential of this method in real-time KPOD, an epoch-wise LS experiment was conducted. The results show that with the help of the RW constraints, not only the positioning accuracy could be improved, but also the converge time could be shorted. All these confirmed the improvement brought by the RW constraints. However, attention needs to be paid to that all the experiments in this study were based on the fact that the GRACE USO shows a character of stable short-term Allan deviation. For other LEOs with different types of on-board clocks, detailed investigations are needed in advance.

## Figures and Tables

**Table 1:** Measurements and error models.

Model	Description
GPS tracking data	Undifferenced Ionosphere-free Code and Phase
GPS orbits	IGS Final Orbits and 30-s Clocks
ERP	IERS 2010
GPS phase model	IGS08.atx (week 1930)
GRACE phase model	Phase Center Offset (level 1B)
Stochastic model	Elevation Dependent Model
Priori coordinates	GFZ NAV1B Products
Priori coordinates constraint	100 m
Priori receiver clock constraint	9000 m
Elevation cutoff	0°
Sampling interval	10 s
Arc coverage	24 h
Ionosphere delay	Ionosphere-free Combination
Phase wind-up	Model [19]
Relativistic corrections for GPS	Shapiro Effect [20] Model [21]
Ambiguity resolution	Uncalibrated Phase Delay Method [22]
Post-processing mode	LS solution
Simulated real-time processing mode	Epoch-wise LS solution

**Table 2:** RMS and improvements, float solution with and without RW constraints, GRACE-A/B, 1–28 February 2007.

	GRACE-A			GRACE-B		
RMS	Radial/cm	Along/cm	Cross/cm	Radial/cm	Along/cm	Cross/cm
PPP float + RW	2.9	2.4	3.2	3.0	2.7	2.7
PPP float	3.6	2.7	3.5	4.4	3.4	3.2
Improvement	19.44%	11.11%	8.57%	31.82%	20.59%	15.63%

**Table 3:** RMS and improvement, PPP-AR solution with and without RW constraints, GRACE-A/B, 1–28 February 2007.

	GRACE-A			GRACE-B		
RMS	Radial/cm	Along/cm	Cross/cm	Radial/cm	Along/cm	Cross/cm
PPP-AR + RW	2.5	1.7	1.0	2.4	1.8	1.1
PPP-AR	3.2	1.8	1.1	3.7	2.2	1.2
Improvement	21.88%	5.56%	9.09%	35.14%	18.18%	8.33%

**Table 4:** RMS and improvement, simulated real time float solution with and without RW constraints, GRACE-A/B, 1 February 2007.

	GRACE-A			GRACE-B		
RMS	Radial/cm	Along/cm	Cross/cm	Radial/cm	Along/cm	Cross/cm
PPP float + RW	3.0	3.2	2.1	3.0	3.3	2.3
PPP float	4.1	4.1	2.5	5.6	5.2	3.4
Improvement	26.83%	21.95%	16.00%	46.43%	36.54%	32.35%

**Table 5:** Residuals in radial, along and cross components, simulated real time float solution with and without RW constraints, GRACE-B, 00:10 and 00:30, 1 February 2007.

Time	Float Residuals			Float + Random Walk Residuals		
	Radial/cm	Along/cm	Cross/cm	Radial/cm	Along/cm	Cross/cm
00:10	−54.7	17.3	−45.5	−14.2	−12.5	19.4
00:30	−8.2	7.0	−8.0	2.2	10.9	−10.3
